# Efficacy and Safety of Serotonin-Norepinephrine Reuptake Inhibitors (SNRIs) in Chronic Pain Conditions Compared with Placebo in Maintaining Functional Improvement and Quality of Life: A Systematic Review

**DOI:** 10.1192/bjo.2025.10789

**Published:** 2025-06-20

**Authors:** Betsy Marina Babu, Gaurav Uppal, Sathyan Soundarajan, Rohan Chauhan, Aisha Mohammed Zein Ali

**Affiliations:** 1 London and KSS School of Psychiatry, London, United Kingdom; 2 Satyam Hospital, Ludhiana, India; 3 Wrexam Maelor Hospital, North Wales, United Kingdom; 4 Fortis Hospital, Ludhiana, India; 5 Alamal Hospital, Dubai, UAE

## Abstract

**Aims:** With this systematic review, an attempt will be made to systematically compare the efficacy and side effects of Serotonin-Norepinephrine Reuptake Inhibitors (SNRIs) in Chronic Pain Conditions in terms of functional improvement and quality of life with placebo.

**Methods:** The relevant studies were included after conducting a literature search in OVID, PubMed/MEDLINE, EMBASE/SIGLE, CINAHL, and Web of Science. Randomized controlled trials that compared SNRIs in chronic pain were included in the review. In the assessment of study quality, the Cochrane Risk-of-Bias tool was used. A number of data extractors and analysts were used in the study so as to reduce possible bias in the study results.

**Results:** Duloxetine appeared to be the SNRI with the best therapeutic efficacy profile across different chronic pain types and aetiologies such as osteoarthritis, fibromyalgia, and chronic low back pain. It showed reasonable effectiveness in alleviating pain magnitude and enhancing motor activity. The results were consistent between standard and higher doses of single administration 60 mg. Duloxetine demonstrated a significant reduction in pain when compared with placebo. An improvement in functional status and quality of life measured through self-reported scales was also noted. This trend was more pronounced in the treatment of the patients on SNRI compared with those on placebo.

**Conclusion:** Duloxetine appears to be effective for chronic pain conditions and may help reduce pain intensity, increase functional status and improve the quality of life. Even though rigorous trials indicate that these drugs are superior to placebo after 8–16 weeks, long-term effect and safety profiles are not very clear, and more long-term studies are needed to understand the durability and application of the drugs in clinical practices. Further research should prospectively investigate the efficacy in long-term pain, different comorbid conditions, and include predictors of SNRI response.

